# Green production efficiency of China’s hog breeding industry: Spatial divergence and its driving factors

**DOI:** 10.1371/journal.pone.0288176

**Published:** 2023-11-03

**Authors:** Yifan Ji, Zejun He, Ningjie Li, Chun Li, Tao Xu

**Affiliations:** 1 International Business School, Hainan University, Haikou, Hainan, China; 2 College of Economic and Management, Henan Agricultural University, Zhengzhou, Henan, China; East China Normal University, CHINA

## Abstract

This paper analyzes the green production efficiency (GPE) and spatial divergence of the hog breeding industry, with the aim of providing a foundation for the rational layout of hog breeding and promoting the industry’s high-quality development. The paper uses the SBM model to estimate GPE in 29 provinces, cities, and districts of China from 2006 to 2019. Furthermore, it analyzes the spatial divergence of GPE and its driving factors using divergence indexes and the Geodetector. The results show that China’s GPE of the hog breeding industry increased from 0.409 to 0.496 between 2006 and 2019. The highest efficiency occurred during the I-period, while the lowest efficiency was observed during the II-period. The highest efficiency was in the key development region, and the lowest efficiency was in the potential growth region. The spatial divergence of GPE in the hog breeding industry expanded, with labor input, non-point source pollution, resource endowment, and environmental load bearing being the main driving factors for the expansion in each period. The potential growth region had the largest spatial divergence, mainly affected by resource endowment. In contrast, the constrained development region had the smallest spatial divergence, mainly affected by resource endowment and pollutant emissions. The spatial divergence of moderate and key development regions was considerable, mainly affected by environmental investment, environmental load bearing, and pollutant emissions. Therefore, the key to improving the GPE of the hog breeding industry is to promote the adoption of advanced technology, such as labor-saving, material-saving, and emission reduction technologies. Moreover, several actions should be taken to reduce the spatial divergence among different regions, such as integrated breeding, clean standards, large-scale breeding, and high-end boutique.

## Introduction

The hog breeding industry plays a crucial role as a core sector and a major component of the livestock industry worldwide. Pork production accounts for 32.57% of the total meat output globally Data source: EPS data platform (https://www.epsnet.com.cn) for 2020 data., with 100.853 million tons of pork consumed worldwide in 2021 Data source: USDA Data (https://www.usda.gov/topics/data)., serving as a significant protein source for human consumption. Nevertheless, hog breeding is a highly polluting industry that faces prominent resource and environmental constraints [1]. On the one hand, as the cost of land, labor, and energy continues to increase, the resource dividend is gradually disappearing. On the other hand, the heavy and difficult-to-treat manure emissions have adverse effects on the ecological environment [2]. Particularly, China, as the largest pork consumer and producer globally, where pork consumption accounts for 44% of the world’s total consumption and production accounts for 38.3% of the total global production Data source: EPS data platform (https://www.epsnet.com.cn) for 2020 data., continues to face increasingly serious environmental challenges while improving pork production efficiency. It is a long way to go to achieve China’s double carbon goal. Therefore, China’s hog breeding industry has reached a crucial stage of high-quality development that demands equal emphasis on both “efficiency” and “decontamination” [3]. The practice of high-quality development in the Chinese hog breeding industry can significantly contribute to the global hog industry’s development and environmental improvement.

As a crucial metric for evaluating industrial efficiency and competitiveness, Green Production Efficiency (GPE) serves as a vital benchmark for gauging the level of high-quality development achieved by the hog breeding industry. GPE is a study of the degree of coordination between economic development and environmental resources, based on the limited environmental carrying capacity, to achieve the goal of energy conservation, consumption reduction, and pollution reduction in the production process, using advanced management methods and technology. It not only evaluates the efficiency of resource inputs, such as land, labor, and capital but also takes environmental factors into account to comprehensively measure the overall efficiency of industries under the constraints of resource and environmental factors [4]. GPE has emerged as an important reference for assessing the status of high-quality industrial development and providing theoretical support and policy guidance for subsequent development [5].

Scholars have shown great interest in GPE, conducting extensive research on its measurement and application, focusing on its levels in regions, cities, enterprises, and industries, as well as various key factors that affect it [6–8]. In terms of specific studies at the industry level, researchers have concentrated on the GPE of highly polluting and socially focused industries such as industry, manufacturing, and agriculture (plantation). However, studies on GPE in the livestock industry are relatively scarce. Only Han et al. [9] measured the environmental TFP of the livestock industry in each province of China and applied spatial econometric models to explore its influencing factors. Zhong et al. [10, 11] measured the GTFP of hens and dairy cows in different scales and regions in China. Research on GPE of hog breeding industry is even rarer. Zhao et al. [12] evaluated the GPE of hog breeding of various scales in 18 provinces of China based on the calculation of pollutant emission, Zhong et al. [13] conducted a detailed analysis of the efficiency of different scale breeding based on the comprehensive evaluation of hog GPE. Although numerous studies have been conducted on GPE in various sectors, the hog breeding industry’s functional areas still lack sufficient research attention, failing to account for the unique GPE characteristics that differ across these areas. Conducting more targeted and detailed research on GPE in the hog breeding industry is crucial to achieving high-quality development in this field.

Due to variations in resource endowments, natural conditions, and economic development levels, the GPE of each functional area in hog breeding differs and exhibits significant spatial divergence. This spatial divergence not only leads to an unbalanced allocation of resources and technologies and a loss of coordination in industrial development, but also affects the effectiveness of emission reduction and environmental management, resulting in social green inequity and hindering high-quality development in the industry [14]. Studies have extensively analyzed the current situation of GPE spatial divergence and evolution at the overall agricultural level, focusing on GPE index divergence and evolutionary trends, and have confirmed the temporal, spatial, and regional variability and clustering characteristics of agricultural GPE changes [5, 15, 16]. However, agricultural GPE is significantly different due to varying development levels and positioning in different economic regions, and shows spatial dependence among regions [17, 18]. Researchers have explored the causes of GPE in each region, focusing on the effects of economic factors such as the level of economic development, agricultural trade, agro-industrial agglomeration, agricultural output growth, and scale level on GPE [19–21]. With the progress of industry and the development of the green concept, attention has gradually shifted to the influence of environmental factors on production efficiency [22, 23]. Existing studies have confirmed the existence of spatial differentiation in agricultural GPE, but the extent of spatial divergence of livestock GPE, such as hog breeding, has received less attention, and insufficient focus has been given to the sources of divergence. Moreover, while the causes of GPE levels and evolution have received sufficient attention, few studies have explored the drivers of GPE spatial divergence from both internal and external perspectives, leading to a lack of basis for coordinated industrial development strategies.

Concluding this paper, statistical data is employed to measure the GPE of the hog breeding industry in 29 provinces of China from 2006 to 2019 based on the SBM model. Additionally, divergence indexes and Geodetector are utilized to explore the spatial divergence characteristics and its driving factors, respectively. Compared to previous research, this paper’s marginal contributions are as follows: firstly, it concentrates on the spatial divergence of GPE in the hog breeding industry and scrutinizes the sources of its spatial divergence, providing a reference basis for the industry’s green and coordinated development. Secondly, it examines the GPE characteristics in different areas based on the division of functional areas of the hog breeding industry to enhance the precision and relevance of research outcomes. Thirdly, the paper analyses the driving factors of spatial divergence of GPE in hog breeding industry from both internal and external perspectives, exploring the causes of its spatial divergence. The study results can serve as a reference for promoting coordinated regional development of the hog breeding industry, and thus improving the quality of its development.

## Methods and materials

This chapter presents a comprehensive account of the methods and data sources employed in the research process of this paper. It elucidates the procedure for choosing indicators to measure GPE in the hog breeding industry, along with the underlying rationale for selecting the drivers. This chapter furnishes methods and material backing for calculating and analyzing the research findings in the subsequent section.

### Data

This study considers 29 provinces, cities, and districts as the DMU for measuring the industrial development pattern influenced by China’s development plan. To ensure comprehensive coverage, the five provinces and districts of Hong Kong, Macao, Taiwan, Tibet, and Ningxia were excluded due to missing data. The sample period is divided into three periods: the I-period (2006~2010), the II-period (2011~2015), and the III-period (2016~2019) Classification basis: the *Eleventh Five-Year Plan for the Development of National Animal Husbandry (2006–2010)*, the *Twelfth Five-Year Plan for the Development of National Animal Husbandry (2011–2015)* and the *National Pig Production Development Plan (2016–2020)* issued by the Ministry of Agriculture of China in 2006, 2011 and 2016, respectively, are long-term plans, which provide for the development of each five-year. The plans lay out the development path of the industry and provide goals and directions for the development vision of the hog breeding industry, which have an important guiding role for the industry. Examining the GPE of the hog breeding industry in different planning periods can reflect the development characteristics of the industry led by policies, so this paper divides the sample period into I-period (2006–2010), II-period (2011–2015) and III-period (2016–2019).

(due to some missing data, the 2020 GPE was not calculated). Data were primarily obtained from the *Compilation of Information on the Cost and Benefit of Agricultural Products in China*, *China Agricultural Statistical Yearbook*, *China Rural Statistical Yearbook*, *China Environmental Statistical Yearbook*, and EPS database from 2005 to 2020. Price-related data were adjusted for inflation using the price index of agricultural production materials (with 2006 as the base period) to ensure accurate comparisons.

Moreover, considering various factors such as product development base, environmental impact, resource allocation, consumer preference, slaughter, and processing, the Ministry of Agriculture has released *the China Pig Production Development Plan (2016–2020)*, which divides the country into four pig development regions: key development region, constrained development region, potential growth region, and moderate development region*China Pig Production Development Plan* (2016–2020) issued by the Ministry of Agriculture and Rural Affairs of the People’s Republic of China divides the pig development regions, with the key development region including Hebei, Shandong, Henan, Chongqing, Guangxi, Sichuan, Hainan; the constrained development region including Beijing, Tianjin, Shanghai, Jiangsu, Zhejiang, Fujian, Anhui, Jiangxi, Hubei, Hunan and Guangdong; the potential growth region including Liaoning, Jilin, Heilongjiang, Inner Mongolia, Yunnan and Guizhou; and the moderate development region including Shanxi, Shaanxi, Gansu, Xinjiang, Tibet, Qinghai, and Ningxia.

. In this paper, we further extend this classification to analyze the spatial divergence and evolution of GPE.

The data utilized in this study are official statistics and do not involve human participants or raise ethical issues. Therefore, this study was not subject to review and approval by an institutional review board (ethics committee), and participant consent was not obtained.

### Variable settings

According to the arrangement of the study, this section selects the input-output variables required to measure GPE and the driving factors variables that may affect the spatial divergence of GPE to provide material support for the study. presented in Table 1.

**Table 1:** Input-output and driving factors variables.

Indicators	Variables	Description of Variables	Reference
**Conventional input**	Labor input	Labor cost per unit (USD)	Zhao et al. [12]
	Piglet input	Piglet cost per unit (USD)	Zhao et al. [12]
	Feed input	Concentrated feed cost per unit (USD)	Zhao et al. [12]
**Resource input**	Energy input	Fuel power cost per unit (USD)	Zhong et al. [11]
	Water input	Feeding water cost per unit (USD)	Zhong et al. [11]
**Desirable output**	Net hog weight gain	Weight of hog product-weight of piglets (kg)	Zhao et al. [12]
**Undesirable output**	Non-point source pollutants Emissions	Non-point source pollutants (10 kt)	Streimikis & Saraji [31]
	Carbon emissions	Carbon emissions (10 kt)	Zhu et al. [24]
**Driving Factors**	Scale breeding	Number of large-scale hog farms/total number of farms (%)	Zhong et al. [11]
	Environmental load bearing	Hog stock/ cultivated land (head/m^2^)	Zheng et al. [25]
	Production operation capability	Number of slaughtered fattened hogs/quantity of live hogs at the beginning of the year (%)	Wang et al. [47]
	Resource endowment	Maize yield (10 kt)	Wang et al. [47]
	Environmental investment	Environmental governance investment (100 million USD)	Wang et al. [47]

#### Input-output variables

When selecting variables, it is recommended to prioritize physical quantities whenever possible [12]. In addition, to ensure scientific and comparable results, it is important to choose variables that are consistently measured across regions and less influenced by market price fluctuations [26]. Regarding input indicators, previous research has typically categorized them into two types: conventional inputs and resource inputs. The key costs of traditional hog breeding include labor, piglets, and feed. Therefore, this study uses labor input, piglet input, and feed input as conventional input variables to measure the production efficiency of the hog breeding industry. To comprehensively evaluate the green production capacity of hog breeding, this study also incorporates water and energy—both closely linked to sustainable development—as input variables. Specifically, water input and energy input are included in the analysis.

The selected desirable output indicator is the net main product yield (net hog weight gain), which is the difference between the main product yield of hogs and the weight of piglets. In regards to the selection of undesirable output indicators, both carbon emissions and non-point source pollutants are deemed as undesirable outputs of hog breeding [27]. While some scholars choose carbon emission [28], others prefer the amount of non-point source pollutants [29]. This paper measures both carbon emissions and non-point source pollutants by drawing on Zhou et al. [30] and Streimikis & Saraji [31]. To accurately estimate GPE, this paper considered as many pollutants as possible, and finally selected COD, total nitrogen (TN), and total phosphorus (TP) as non-point source pollutants. The pollutants emitted from the hog breeding process were then converted into carbon equivalents to measure their total carbon emissions.

According to the study by Zhang et al. [32], the emissions of non-point source pollutants can be calculated by the following formulas:

$$COD=N\times T\times (G_{m}\times G_{m-COD}+G_{u}\times G_{u-COD})$$ (1)

$$TN=N\times T\times (G_{m}\times G_{m-TN}+G_{u}\times G_{u-TN})$$ (2)

$$TP=N\times T\times (G_{m}\times G_{m-TP}+G_{u}\times G_{u-TP})$$ (3)

where, *COD*, *TN*, *TP* represent COD, TN and TP emission, *N* stands for hog breeding quantity, measured by annual hog output, *T* represents feeding cycle, *G*_*m*_ and *G*_*u*_ represent daily emission coefficient of hog manure and hog urine respectively, *G*_*m-COD*_ and *G*_*u-COD*_ represent COD emission coefficient of hog manure and hog urine respectively, *G*_*m-TN*_ and *G*_*u-TN*_ represent TN emission coefficient of hog manure and hog urine, *G*_*m-TP*_ and *G*_*u-TP*_ represent TP emission coefficient of hog manure and hog urine.

Referring to Yao et al. [33], the carbon emissions from hog breeding are measured based on the whole life cycle with the following equations:

$$TC_{sc}=APP\times (\frac{cost_{e}}{price_{e}}\times ef_{e}+\frac{cost_{c}}{price_{c}}\times ef_{c})$$ (4)

$$TC_{sg}=P\times \frac{MJ}{e}\times ef_{e}$$ (5)

$$APP=T\times \frac{N}{365}$$ (6)

where, *TC*_*sc*_ and *TC*_*sg*_ represent the carbon emission of feeding process and processing process respectively, *APP* is the average annual feeding quantity of hogs, *cost*_*e*_ and *cost*_*c*_ represent the electricity expenditure and coal expenditure per unit of hog breeding respectively, *price*_*e*_ and *price*_*c*_ represent the unit price of electricity and coal respectively, *ef*_*e*_ and *ef*_*c*_ are the CO_2_ emission coefficient of electricity consumption and coal consumption. *P* represents the annual output of pork, *MJ* is the energy dissipation coefficient of pork per unit processing, and *e* is the heat value generated by consuming one unit of electricity.

$$TC_{CH_{4}}=APP\times (ef_{i-CH_{4}}+ef_{j-CH_{4}})\times GWP_{CH_{4}}$$ (7)

$$TC_{N_{2}O}=APP\times ef_{j-N_{2}O}\times GWP_{N_{2}O}$$ (8)

$$TC=(TC_{sc}+TC_{sg}+TC_{CH_{4}}+TC_{N_{2}O})\times e_{tpf}$$ (9)

where, $TC_{CH_{4}}$, $TC_{N_{2}O}$ and *TC* represent CH_4_ global warming potential, N_2_O global warming potential and total carbon emission of hog breeding industry respectively, $ef_{i-CH_{4}}$, $ef_{j-CH_{4}}$ and $ef_{j-N_{2}O}$ represent CH_4_ emission coefficient of pig gastrointestinal fermentation, CH_4_ and N_2_O emission coefficient of manure management respectively. $GWP_{CH_{4}}$ and $GWP_{N_{2}O}$ are CH_4_ and N_2_O global warming potential respectively, and *e*_*tpf*_ is the conversion of CO_2_ equivalent is converted to standard carbon coefficient. Table 2 shows the meaning of various emission coefficients and their values.

**Table 2:** Hog breeding pollutant emission coefficient.

	Emission coefficient	Symbol	Value	Units
**Non-point source pollutants emission coefficients**	Feeding cycle	T	199	d
	Daily emission coefficient of hog manure	G_m_	2.00	kg/d
	Daily emission coefficient of hog urine	G_u_	3.30	kg/d
	COD emission coefficient of hog manure	G_m-COD_	52.00	kg/t
	TN emission coefficient of hog manure	G_m-TN_	5.88	kg/t
	TP emission coefficient of hog manure	G_m-TP_	3.41	kg/t
	COD emission coefficient of hog urine	G_u-COD_	9.00	kg/t
	TN emission coefficient of hog urine	G_u-TN_	3.30	kg/t
	TP emission coefficient of hog urine	G_u-TP_	0.52	kg/t
**Carbon emission coefficients**	Unit price of electricity	Price_e_	0.062	USD/kW·h
	Unit price of coal	Price_c_	116	USD/t
	CO_2_ emission coefficient of electric consumption	ef_e_	0.973	tCO2/MW·h
	CO_2_ emission coefficient of coal consumption	ef_c_	1.98	t/t
	Energy dissipation coefficient per unit processing of pork	MJ	3.76	MJ/kg
	Heat value generated by consuming one unit of electricity	e	3.60	MJ
	CH_4_ emission coefficient of hog gastrointestinal fermentation	ef_i-CH4_	1.00	kg/ head
	CH_4_ emission coefficient of hog manure management	ef_j-CH4_	3.50	kg/ head
	N_2_O emission coefficient of hog manure management	ef_j-N2O_	0.530	kg/ head
	CH_4_ global warming potential	GWP_CH4_	21	
	N_2_O global warming potential	GWP_N2O_	310	
	CO_2_ equivalent is converted to standard carbon coefficient	e_tpf_	0.2728	

Note: The emission coefficient of non-point source pollutants is taken from the statistics released by the Ministry of Ecology and Environment of China (https://www.mee.gov.cn). Carbon emission coefficient is determined in conjunction with IPCC guidelinesIPCC. IPCC Guidelines for National Greenhouse Gas Inventories Volume 4: Agriculture, Forestry and Other Land Use[R]. Geneva: IPCC, 2006

#### Driving factors variables

This paper has selected several independent variables, including scale breeding, environmental load bearing, production and operation capacity, resource endowment, and environmental investment, to investigate their impact on the spatial divergence of GPE in hog breeding. The scale of hog breeding is closely linked to its standardization capacity, which develops in tandem [34]. Standardization is a vital prerequisite for achieving green and high-quality development in the hog breeding industry. Hence, scale breeding is a crucial factor affecting GPE in this industry, and differences in scale breeding across regions will exacerbate the spatial divergence in GPE.

The environmental load bearing refers to the number of hogs per unit of arable land, and mitigating the environmental carrying capacity is a crucial measure for improving the industry’s green efficiency and sustainable development [35]. The higher the regional environmental loading intensity, the greater the number of hogs bred, the more significant the impact on breeding activities and the level of environmental management. Therefore, environmental load bearing is an important variable included in the system of driving factors.

The hog output rate, a pivotal indicator of hog production and capacity [36], is a significant reflection of the industry’s production and operational capacity. Developing production and operational capacity constitutes an effective approach to enhance the industry’s green development capacity [37]. Consequently, the variation in production and operation capacity emerges as a crucial factor contributing to the spatial divergence in GPE of the hog breeding industry.

Resource endowment exerts a direct and positive influence on the industry’s green development efficiency [38] and constitutes the fundamental basis for enhancing the GPE of the hog breeding industry. The differences in resource endowments among regions may lead to divergences in GPE. Additionally, since maize is the primary raw material for hog feed, a higher maize yield corresponds to stronger green farming capacity for hogs. Thus, this paper employs Kolleen & Norman’s [39] methodology to gauge feed production by maize yield, which in turn represents the resource endowment status of the hog farming industry in each province, and scrutinizes its impact on the spatial divergence of GPE.

The effectiveness of environmental management practices is heavily influenced by the level of environmental investment made in the region, with higher investments indicating a greater emphasis on environmental management [40]. This highlights the importance of promoting green hog breeding practices, which can significantly enhance the industry’s GPE. As a result, differences in the environmental management status of each region can impact the spatial divergence of the hog breeding industry’s GPE across regions.

### Methods

This section provides a detailed description of the methods to be applied in this study, including the SBM model, the divergence index, and the Geodetector, which helps to improve the applicability of the study.

#### SBM model

To measure the GPE of the hog breeding industry in each province, the SBM model based on undesirable outputs is utilized. The non-radial and non-angular SBM directional distance function, proposed by Tone [41], fully addresses the input-output slackness problem. This method directly incorporates slack variables into the objective function to solve the non-zero input-output slackness issue, which eliminates the non-efficiency factors caused by slackness. Furthermore, the SBM model is dimensionless and non-angular, thus avoiding bias and effects of different magnitudes and angle selection differences. The SBM model considers *m* inputs (x), *n*_1_ desirable outputs (*y*^*g*^), and *n*_2_ undesirable outputs (*y*^*b*^) and can be expressed as:

$$Min\rho =\frac{1-\frac{1}{m}\sum_{i=1}^{m}\frac{s_{i}^{-}}{x_{i0}}}{1+\frac{1}{n_{1}+n_{2}}\left(\sum_{r=1}^{n1}\frac{s_{r}^{g}}{y_{r0}^{g}}+\sum_{r=1}^{n2}\frac{s_{r}^{b}}{y_{r0}^{b}}\right)}$$ (10)

$$s.t.\left\{\begin{array}{l} x_{0}=X\lambda +s^{-}\\ y_{0}^{g}=Y^{g}\lambda -s^{g}\\ y_{0}^{b}=Y^{b}\lambda +s^{b}\\ \lambda \geq 0,s^{-}\geq 0,s^{g}\geq 0,s^{b}\geq 0, \end{array}\right.$$ (11)

where *s* represents the slack in inputs and outputs, *s*^-^ indicates too many inputs, *s*^*b*^ indicates too many undesirable outputs, *s*^*g*^ indicates insufficient desirable outputs, *λ* indicates the weights, *ρ*(0≤ *ρ* ≤1) indicates the attainment efficiency score, and the rates of *s*^-^, *s*^*b*^ and *s*^*g*^ are strictly decreasing. When *s*^-^, *s*^*b*^ and *s*^*g*^ are all equal to 0, that is, when *ρ=1*, it means that there is no excess of inputs and undesirable outputs, and there is no deficit of desirable outputs. Thus, the DMU is completely efficient. However, when *s*^-^, *s*^*b*^ and *s*^*g*^ are all greater than 0, that is, when *ρ*<1, it means that there is an efficiency loss in DMU. The output level can be maintained by reducing the input and undesirable output, indicating that the DMU is invalid.

#### Divergence index

The measurement of regional divergence in development levels is commonly done using methods such as the Gini coefficient, the Theil index, and the mean log deviation (generalized entropy index). These methods are sensitive to changes in high, medium, and low levels, respectively, and their results are complementary. Thus, scholars usually analyze regional divergence in development levels based on the comparison results of these three methods [42, 43].

The modified Gini coefficient is widely used in industrial economics and is one of the main methods for measuring differences in the level of industrial development. There are various methods for measuring the Gini coefficient, and this paper adopts the method proposed by Mookherjee and Shorrocks [44]. The basic formula for this method is:

$$GINI=\frac{1}{2n^{2}\mu }\sum \left|GPE_{i}-GPE_{j}\right|$$ (12)

where *n* denotes the number of provinces, *GPE*_*i*_ and*GPE*_*j*_ represent the GPE of the hog breeding industry in province *i* and province *j*, respectively, and *μ* is the average value of GPE in each province.

The basic formulae for the mean log deviation (*GE*_0_) and the Thiel index (*GE*_1_) are as follows:

$$GE_{0}(GPE)=\frac{1}{n}\sum {}_{i\in N}ln\frac{\mu }{GPE_{i}}$$ (13)

$$GE_{1}(GPE)=\frac{1}{n}\sum {}_{i\in N}\frac{GPE_{i}}{\mu }ln\frac{GPE_{i}}{\mu }$$ (14)

where *n* denotes the number of provinces, *μ* is the average value of GPE of the hog breeding industry in each province and *GPE*_*i*_ denotes the level of GPE in province *i*.

To explore the trend of inter-provincial differences over time, this paper further examines the convergence of GPE. α convergence is used to analyze the discrete trends in GPE of the national and regional hog breeding industry. The α convergence formula is:

$$\alpha_{t}=\sqrt{n^{-1}\sum_{i=1}^{n}{\left\{GPE_{i}\left(t\right)-\left[n^{-1}\sum_{m=1}^{n}GPE_{m}\left(t\right)\right]\right\}}^{2}}$$ (15)

where *GPE*_*i*_(*t*) denotes the GPE of the hog breeding industry in period *t* of the *i* province, *GPE*_*m*_(*t*) denotes the GPE in period *t* of the *m* province, and *n* denotes the number of provinces. The decreasing value of *α*_*t*_ indicates that the GPE in the sample period is converging and the difference between provinces is narrowing, while the increasing of *α*_*t*_ indicates divergence, and the difference in GPE among provinces is enlarged.

#### Geodetector

The Geographic comprises of four key components, namely the factor detector, interaction detector, risk detector, and ecological detector. The fundamental assumption of this approach is that the spatial distribution of the driving factors responsible for the change is in agreement with the spatial distribution of that particular phenomenon. It suggests that the driving factors significantly impact the spatial divergence of the phenomenon under study [45]. This study investigates the driving factors of spatial divergence of GPE through factor detector and interaction detector. The factor detector is utilized to examine the impact of a factor on the spatial divergence of a variable, whereas the interaction detector is employed to explore the effect of factor interactions on the variable. By developing a novel spatial layer comprising two driving factors, the interaction detector assesses the impact of two factors and their superimposed layers on spatial divergence. The primary aim of the interaction detector is to unveil the interaction between different factors on spatial divergence and to compare the interaction effect with that of a single factor on the variables. The factor detector assesses whether factors affect the spatial divergence of variables by comparing whether the spatial distribution of driving factors and variables is consistent. The degree of factor detector is measured using the q-statistic, expressed as:

$$q=1-\frac{\sum_{h=1}^{H}N_{h}\sigma_{h}^{2}}{N\sigma^{2}}$$ (16)

*q*-statistic denotes the role of factors driving the spatial divergence of GPE, with q ranging from 0 to 1. *N* denotes the regional sample size. *H* is the partition of factors and variables and denotes the sample size of sub-region *h*. *q* = 0 means that the spatially stratified heterogeneity is not influenced by the factor, and *q* = 1 means that the spatial divergence is completely influenced by the factor. The larger the *q*-statistic, the greater the influence of the factor on the spatial divergence of GPE. In this regard, the *q*-statistic indicates that the spatial divergence of factors explains the spatial divergence of variables, and it does not require a significance test [46]. To process data, all driving factors must be transformed into quantitative variables. This study uses the Q-type clustering analysis method to cluster independent variables into six categories according to numerical size, and observations are grouped into six types from high to low. Finally, the Geodetector is used to calculate the degree of influence of each factor on the spatial divergence of GPE in the hog breeding industry.

## Results

This chapter employs the aforementioned materials and methods to gauge the GPE of the hog breeding industry and conducts an analysis of the outcomes. It expounds upon the development characteristics and evolutionary trends of GPE in diverse regions and provinces across various time periods, examining both spatial and temporal dimensions, while offering profound interpretations for the underlying reasons. The findings of this chapter serve as the foundation for scrutinizing the spatial divergence of GPE and its driving factors.

### Statistical analysis of variables

Table 3 presents the results of the descriptive statistical analysis of input and output variables. The analysis revealed that, firstly, conventional input variables witnessed a significant increase as compared to 2006, while resource input variables remained stable, and the net weight gain of hogs also increased significantly. This suggests that hog production capacity increased with the increase in resource input. Secondly, there was a significant decrease in non-point source pollutant emissions, whereas carbon emissions increased initially and then decreased, indicating the considerable effect of pollutant emission reduction during the hog breeding process.

**Table 3:** Descriptive statistics of input-output variables.

Year	Labor input (USD)	Piglet input (USD)	Feed input (USD)	Energy input (USD)	Water input (USD)	Net hog weight gain(kg)	COD (billion tons)	TD (billion tons)	TP (billion tons)	Carbon emission (billion tons)
2006	4.559	24.258	54.878	0.892	0.233	81.3	0.134	0.023	0.0086	10.51
2007	5.894	43.353	63.409	0.906	0.281	84.2	0.132	0.022	0.0084	9.65
2008	6.106	58.074	73.636	0.955	0.295	86.7	0.117	0.020	0.0075	11.73
2009	8.412	51.501	95.254	1.162	0.344	88.11	0.123	0.021	0.0079	12.43
2010	9.253	46.309	100.219	1.080	0.344	89.64	0.125	0.021	0.0080	12.19
2011	11.344	70.028	108.882	1.076	0.341	91.51	0.124	0.021	0.0079	12.79
2012	13.304	78.966	128.491	1.152	0.369	93.65	0.124	0.021	0.0079	13.68
2013	15.764	78.554	142.222	1.146	0.394	93.86	0.127	0.021	0.0081	13.66
2014	16.442	73.195	149.258	1.291	0.431	95.81	0.126	0.021	0.0081	14.98
2015	16.412	77.212	138.169	1.217	0.417	96.31	0.124	0.021	0.0079	14.97
2016	16.445	107.976	125.459	1.153	0.394	98.86	0.120	0.020	0.0077	14.69
2017	16.079	95.468	120.178	1.194	0.401	99.91	0.116	0.020	0.0074	16.34
2018	16.218	70.318	123.615	1.230	0.428	101.56	0.117	0.020	0.0075	16.53
2019	15.960	92.559	122.414	1.135	0.399	105.47	0.114	0.019	0.0073	13.46

### Measurement of the GPE

**Fig 1:**
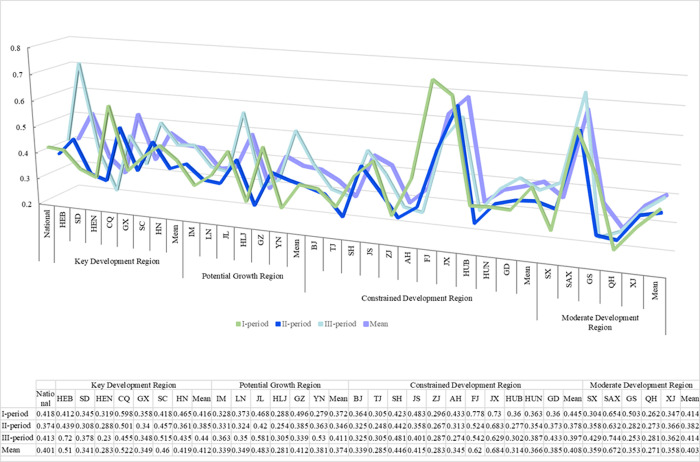
GPE of the hog breeding industry. Note: HEB, SD, HEN, CQ, GX, SC, HN, IM, LN, JL, HLJ, GZ, YN, BJ, TJ, SH, JS, ZJ, AH, FJ, JX, HUB, HUN, GD, SX, SAX, GS, QH, XJ respectively stand for Hebei province, Shandong province, Henan province, Chongqing, Guangxi province, Sichuan province, Hainan province, Inner Mongolia province, Liaoning province, Jilin province, Heilongjiang province, Guizhou province, Yunnan province, Beijing, Shanghai, Jiangsu province, Zhejiang province, Anhui province, Fujian province, Jiangxi province, Hubei province, Hunan province, Guangdong province, Shanxi province, Shaanxi province, Gansu province, Qinghai province, and Xinjiang province.

The SBM model was used to calculate the mean values of GPE in the hog breeding industry in the key development region, constrained development region, potential growth region, and moderate development region in the I, II, and III periods. The results are shown in Fig 1.

As shown in Fig 1, the overall GPE increased to an average of 0.401, indicating an improvement in the industry’s efficiency. The highest GPE was observed in the I-period, followed by a decrease in the II-period, and a recovery in the III-period. Regionally, the key development region had the highest overall GPE of 0.412, followed by the constrained and moderate development regions, and the potential growth region had the lowest overall GPE of 0.374. In addition, except for the constrained development region, the GPE of the remaining regions grew during the sample period, with the fastest growth rate in the potential growth region, with an average GPE of 0.411 in the III-period, an increase of 10.5% compared to the I-period. Followed by the key development region, with an average 5.8% increase in GPE, and the moderate development region was stable overall, while the constrained development region decreased by 10.8% in GPE. Nine provinces, including Beijing, Tianjin, Shanxi, Inner Mongolia, Liaoning, Jilin, Shanghai, Guangxi, and Gansu, experienced a decline in GPE throughout the sample period, while the remaining 20 provinces showed an increase. Most provinces exhibited an increase in GPE in the I-period and III-period, and a decrease in the II-period.

**Fig 2:**
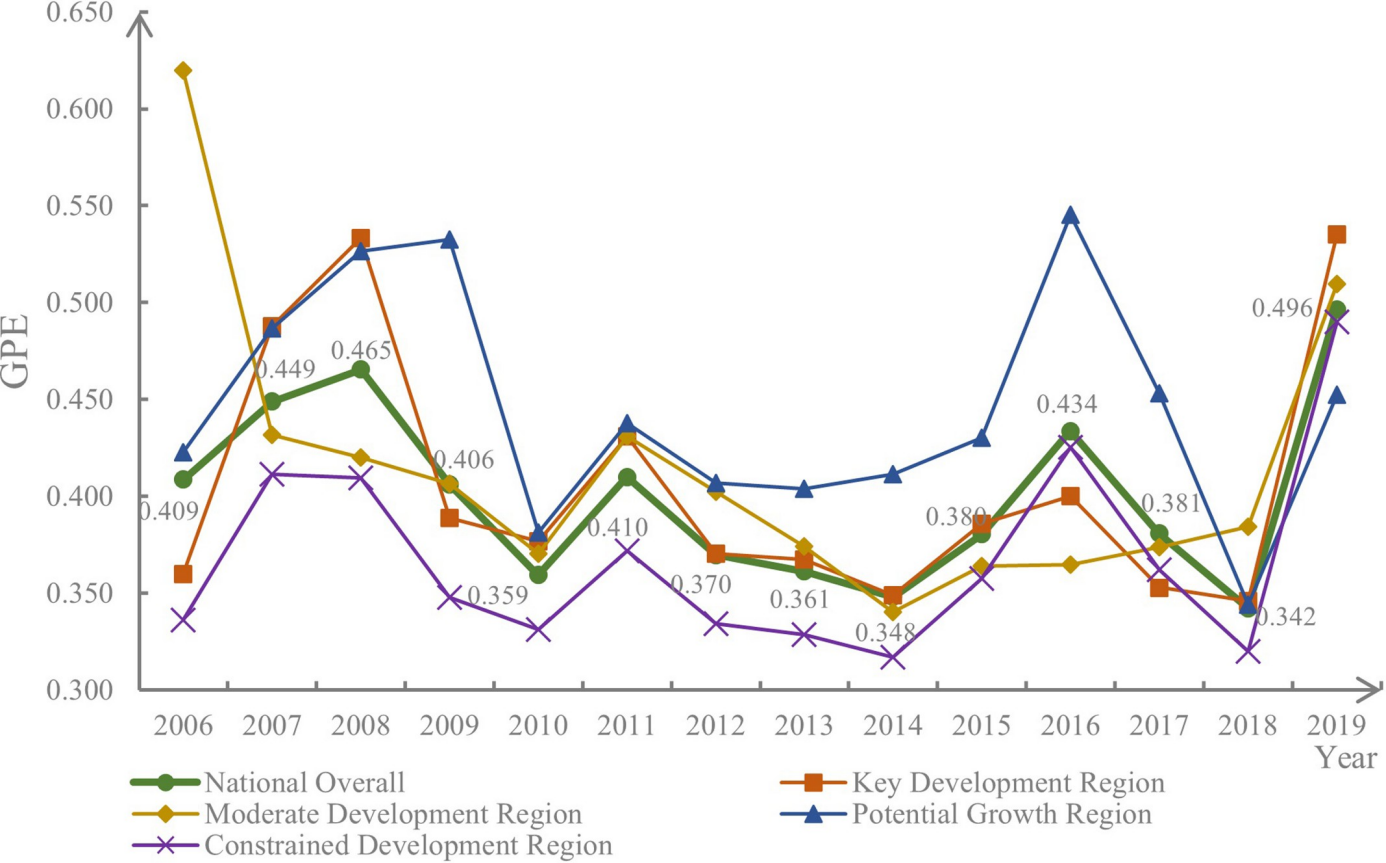
Temporal evolution of GPE in the hog breeding industry.

Fig 2 depicts the chronological progression of the GPE in China’s hog breeding industry. Nationally, the GPE observed a substantial increase from 0.409 to 0.496. GPE displayed an initial incline and subsequent decline during the I-period, reaching its highest value in 2008. The cause was mainly attributed to the outbreak of Porcine Reproductive and Respiratory Syndrome (PRRS) in hogs from 2007 to 2008, leading to a persistent decline in hog population and a consequent decrease in hog production capacity. During the II-period, GPE experienced a slight drop between 2011 and 2013, followed by a gradual improvement in 2014. The main reason for the decline was the lean meat powder scandal in 2011, which affected the hog production efficiency. However, the *Regulations on Prevention and Control of Pollution in Livestock and Poultry Scale Farming* promulgated in 2014 stimulated the green development of the hog breeding industry, thereby leading to the recovery of GPE. During the III-period, GPE witnessed an initial decline and subsequent increase, attaining its lowest point of 0.342 in 2018, followed by a sharp rise in 2019. This was primarily due to the impact of African Swine Fever (ASF) in 2018, which significantly reduced hog breeding efficiency and spurred the rapid reorganization of the industry, culminating in the enhanced green development of the hog breeding industry and a corresponding surge in hog GPE in 2019.

At the regional level, the GPE in the potential growth region exhibited cyclical fluctuations with a period of five years, and had the highest value overall. The GPE first increased and then decreased in the I-period, remained stable during the II-period, and increased again in the III-period. Due to the abundance of resources in the region, the production efficiency of hog breeding was high, and showed an upward trend. In the moderate development region, the GPE experienced a rapid decline from 2006 to 2007, followed by a sharp increase from 2018 to 2019, with a relatively stable period in between. This region experienced less breeding pressure and had a high level of green development overall, with little impact from the hog breeding industry on its economic development and market stability. In the key development region, the GPE of the hog breeding industry first increased and then decreased in the I-period, remained relatively stable during the II-period, and experienced a decrease followed by an increase in the III-period. This region had a large proportion of the hog breeding industry, and was greatly influenced by animal diseases and government regulations, resulting in a fluctuating trend. The overall GPE of hog breeding in the constrained development region was the lowest, with a fluctuating trend in development. This region was economically developed, with a dense water network and limited hog breeding, resulting in little influence from various factors on the GPE.

### Spatial divergence

Building upon the aforementioned measurement results, this section proceeds to delve into an in-depth analysis of the extent of divergence in GPE within various regions and provinces throughout the sample period. Expanding on this foundation, the dispersion trend of GPE divergence among different regions is explored through the implementation of an α convergence test, while elucidating the underlying reasons behind this evolving trend. Moreover, this chapter examines the distinctive features of spatial divergence in GPE within the hog breeding sector across different periods and regions, affirming the driving factors through the scrutiny of input-output factors and external factors.

In this paper, Gini coefficient, Theil index and mean log deviation were used to explore the divergence of hog breeding industry GPE in different regions during the sample period. The results are shown in Table 4.

**Table 4:** Divergence of GPE in the hog breeding industry.

Year	GINI	GE_0_	GE_1_	Key Development Region GINI	Moderate Development Region GINI	Potential Growth Region GINI	Constrained Development Region GINI
**2006**	0.107	0.078	0.089	0.034	0.145	0.114	0.024
**2007**	0.089	0.051	0.054	0.054	0.080	0.143	0.055
**2008**	0.101	0.065	0.070	0.105	0.077	0.138	0.053
**2009**	0.091	0.058	0.065	0.077	0.078	0.133	0.033
**2010**	0.061	0.023	0.023	0.062	0.060	0.062	0.042
**I-period**	0.090	0.055	0.060	0.066	0.088	0.118	0.041
**2011**	0.074	0.035	0.036	0.062	0.073	0.090	0.052
**2012**	0.073	0.035	0.036	0.057	0.086	0.088	0.053
**2013**	0.070	0.032	0.034	0.059	0.082	0.089	0.043
**2014**	0.081	0.042	0.045	0.058	0.088	0.111	0.051
**2015**	0.079	0.040	0.040	0.051	0.107	0.086	0.061
**II-period**	0.075	0.037	0.038	0.057	0.087	0.093	0.052
**2016**	0.102	0.069	0.071	0.055	0.126	0.116	0.083
**2017**	0.087	0.049	0.051	0.074	0.086	0.092	0.076
**2018**	0.076	0.037	0.038	0.062	0.094	0.079	0.060
**2019**	0.108	0.075	0.076	0.071	0.131	0.110	0.107
**III-period**	0.093	0.058	0.059	0.066	0.109	0.099	0.082
**Average 1**	0.086	0.049	0.052	0.063	0.094	0.104	0.057
**Average 2**	0.072	0.033	0.035	0.055	0.083	0.089	0.051

Note: average 1 is the mean value of the three indicators from 2006 to 2019, and average 2 is the value of the three indicators measured based on the mean value of GPE in each province from 2006 to 2019.

The Gini coefficient, Thiel index, and mean log deviation all displayed a consistent pattern, revealing that the spatial divergence of GPE initially decreased and then widened, ultimately leading to an overall expansion. The I-period demonstrated a conspicuous trend of narrowing spatial divergence, with the values of the three indicators dropping from 0.107, 0.078, and 0.089 in 2006 to 0.061, 0.023, and 0.023 in 2010. The spatial divergence experienced a slight increase and remained relatively stable in the II-period. In the III-period, the spatial divergence initially decreased and then increased, reaching its lowest point in 2018 with values of 0.076, 0.037, and 0.038 for the three indicators, and then rapidly expanding in 2019. The international community generally regards a Gini coefficient of 0.3 or less as indicating a small gap in the level of inequality. The results demonstrate that, although China’s hog breeding industry’s GPE did not significantly vary from 2006 to 2019, the inter-provincial spatial divergence exhibited a fluctuating trend.

Table 4 examines the spatial divergence of GPE across regions during the sample period, using the Gini coefficient as a measure. Looking at the mean values, the potential growth region had the highest spatial divergence, with mean values of 0.104 and 0.089, followed by the moderate development region and key development region, while the constrained development region had the lowest spatial divergence. In terms of changes in different regions, the overall spatial divergence of GPE remained stable in the key development region and continued to increase in the constrained development region. The spatial divergence in the moderate development region remained stable during the I-period and II-period but increased rapidly during the III-period. In the potential growth region, the spatial divergence decreased initially and then increased, with the smallest spatial divergence in the II-period. It can be observed that the divergence in the green production capacity of hogs between provinces within each region has expanded.

**Fig 3:**
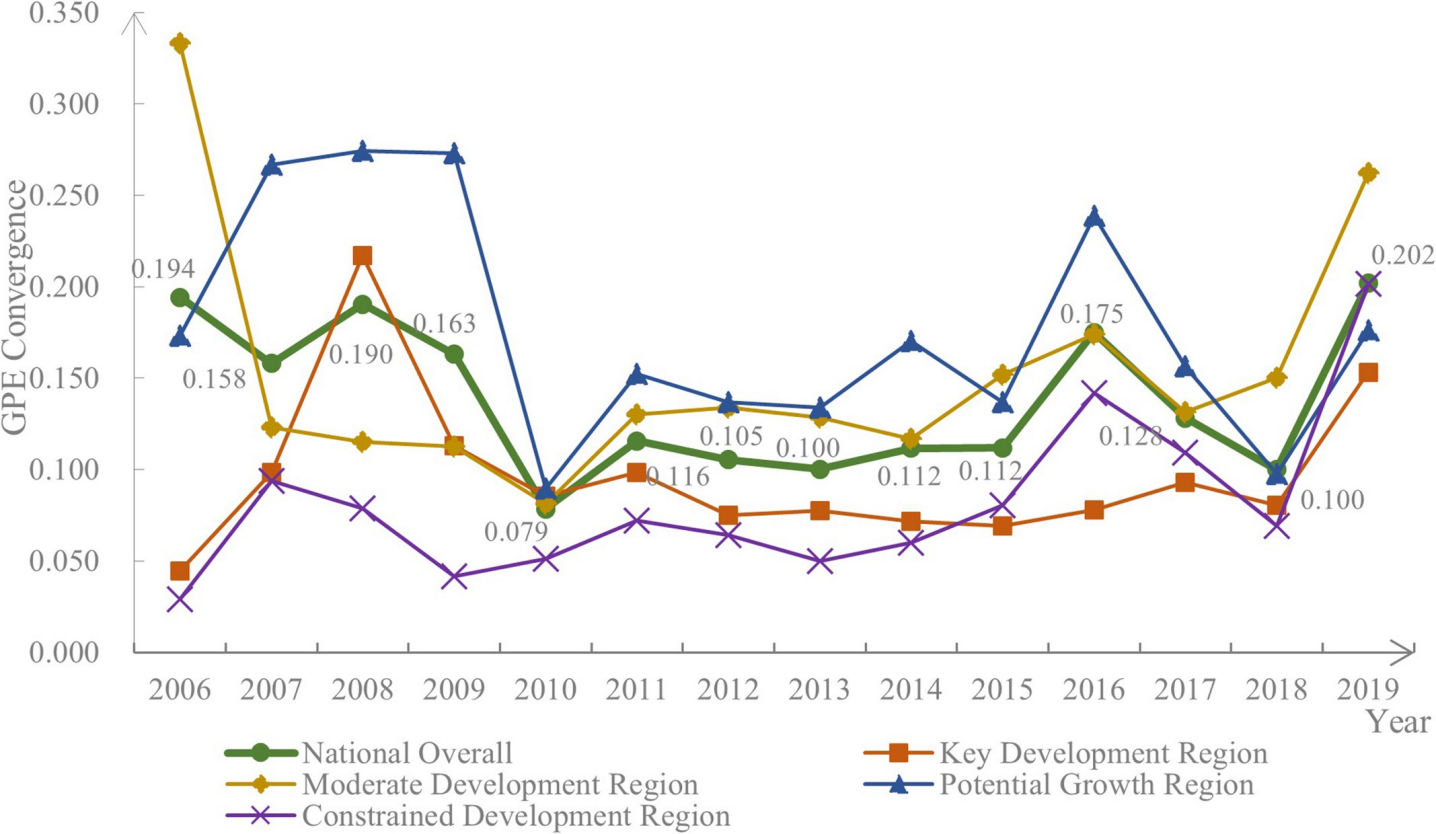
Convergence trend of GPE in the hog breeding industry.

Fig 3 depicts the discrete trends of the spatial divergence of GPE nationwide, using the Gini coefficient as an example, with the α convergence test. The spatial divergence of GPE in China displays α discrete evolution. Except for the moderate development region, which displays convergence evolution as a whole, the α-convergence values of all other regions expand, indicating α discrete evolution. The potential growth region registers the largest value of α but the slowest convergence rate, whereas the constrained development region has the smallest value of α but the fastest convergence rate. The convergence trend varies across periods. In the I-period, the spatial divergence of GPE in the moderate development and potential growth regions displays α discrete, while the key development and constrained development regions show α convergence. During the II-period, the regions remain stable at a lower level of convergence, without significant fluctuations of spatial divergence. In the III-period, the moderate development, potential growth, and constrained development regions evolve in convergence, followed by discretization, whereas the key development region continues in a discrete trend.

Furthermore, the significant fluctuations in convergence during the years 2010, 2016, and 2018 were linked to the adjustments made in industrial development efforts across each region. Between 2008 and 2009, the Chinese government strongly advocated for rural environmental protection and promoted large-scale hog breeding, which led to an increase in the green production capacity of hogs in each province and a reduction in the gap between them in 2010. In 2014, the implementation of the *Regulation on the Prevention and Control of Pollution from Large-scale Breeding of Livestock and Poultry* prompted provincial governments to adjust the direction of industrial development. However, due to differences in the provincial baseline and response measures, spatial divergence in GPE among provinces in the hog breeding industry continued to expand. In 2018, the outbreak of African Swine Fever (ASF) had a severe impact on the hog breeding industry across all regions, leading to a decline in capacity and a reduction in the production gap, resulting in spatial divergence evolving into convergence. Although production capacity recovered after 2018, the direction and strength of adjustments made in the hog industry varied among provinces, which ultimately led to the expansion of spatial divergence.

### Driving factors of the spatial divergence

GPE can be broken down into the productivity of various inputs and outputs, and differences in regional inputs and outputs may impact the spatial divergence of GPE. Additionally, environmental investments, resource endowments, environmental load bearing, scale breeding, and production operation capacity may also have an effect on spatial divergence. This study employs factor detector and interaction detector techniques of Geodetector to examine the influence of constituent and external factors on the spatial-temporal divergence of GPE.

#### Driving factors of the spatial divergence across periods

Tables 5 and 6 present the outcomes of the factor detector and interaction detector analyses regarding the impact of input-output factors on the spatial divergence of GPE. The factor detector reveals that non-point source pollutants and labor inputs were the primary drivers of spatial divergence in GPE throughout the sample period. During the I-period, feed inputs and non-point source pollutants played significant roles in spatial divergence. In the II-period, spatial divergence in GPE primarily stemmed from labor inputs, carbon emissions, and non-point source pollutants. In the III-period, labor inputs and non-point source pollutants emerged as the main driving factors of spatial divergence.

**Table 5:** Effect of input-output on spatial divergence of GPE across periods.

Periods	Labor input	Piglet input	Feed input	Water input	Energy input	Net Output	Carbon Emission	Pollutant emission
**Overall**	0.112	0.044	0.013	0.079	0.030	0.023	0.067	0.113
**I-period**	0.055	0.085	0.091	0.070	0.033	0.038	0.085	0.193
**II-period**	0.288	0.117	0.026	0.124	0.080	0.038	0.164	0.133
**III-period**	0.140	0.085	0.053	0.093	0.071	0.066	0.043	0.133

**Table 6:** Interactive effect of input-output on spatial divergence of GPE across periods.

Periods	Leading interaction factor	q-statistic	Interaction
**Overall**	Labor input ∩ Pollutant emission	0.293	Enhancement
	Piglet input ∩ Carbon Emission	0.292	Enhancement
**I-period**	Feed input ∩ Pollutant emission	0.506	Enhancement
	Piglet input ∩ Pollutant emission	0.491	Enhancement
**II-period**	Piglet input ∩ Carbon Emission	0.614	Enhancement
	Labor input ∩ Pollutant emission	0.588	Enhancement
**III-period**	Piglet input ∩ Water input	0.529	Enhancement
	Piglet input ∩ Net Output	0.506	Enhancement

The interaction detector demonstrates that the combined effect of two factors on GPE exceeded that of individual factors. Among the interactions, the combination of labor inputs and non-point source pollutants had the most substantial impact on spatial divergence of GPE, with a value of 0.293. In the I-period, the interaction between feed inputs and non-point source pollutants had the greatest influence, reaching a value of 0.506. In the II-period, the interaction between piglet inputs and carbon emissions had the largest effect, reaching a value of 0.614. In the III-period, the interaction between piglet inputs and water input had the most significant impact, with a value of 0.529.

These findings indicate a decreasing influence of feed inputs and carbon emissions on the spatial divergence of GPE, while the impact of labor inputs, energy inputs, and net output on spatial divergence has gradually increased. The interactions between input factors consistently displayed a stronger effect, with the interaction between labor inputs and non-point source pollutants exhibiting a growing influence.

The impact of external factors, including environmental investment, resource endowment, environmental load bearing, scale breeding, and production operation capacity, on the GPE of the hog breeding industry is noteworthy. Tables 7 and 8 present the test results of the factor detector and interaction detector for each period. The factor detector indicates that resource endowment had the greatest influence on the spatial divergence of GPE, but its impact decreased over time, with q-statistic decreasing from 0.293 in the I-period to 0.153 in the III-period. This was followed by environmental load bearing, which saw a decrease in q-statistic from 0.091 to 0.076, and scale breeding, which experienced a decrease in q-statistic from 0.149 to 0.030. Meanwhile, the influence of environmental investment and production operation capacity on the spatial divergence decreased to 0.036 and 0.047, respectively, in the III-period. Resource endowment and environmental load bearing were the most important driving factors for the spatial divergence of GPE in the III-period, followed by production operation capacity, environmental investment, and scale breeding. The interaction detector shows that the interaction between all factors in each period had a greater impact than the effect of single factors. The interaction between resource endowment and environmental load bearing had the highest driving value of 0.388 across all periods. It can be concluded that resource endowment and environmental load bearing were the most important factors for the spatial divergence of GPE among regions, leading to the expansion of the spatial divergence of GPE.

**Table 7:** Effect of external factors on spatial divergence of GPE across periods.

Periods	Environmental Investment	Resource Endowment	Environmental Load Bearing	Scale Breeding	Production Operation
**Overall**	0.007	0.247	0.073	0.065	0.007
**I-period**	0.030	0.293	0.091	0.149	0.016
**II-period**	0.018	0.426	0.222	0.165	0.062
**III-period**	0.036	0.153	0.076	0.030	0.047

**Table 8:** Interactive effect of external factors on spatial divergence of GPE across periods.

Periods	Leading interaction factor	q-statistic	Interaction
**Overall**	Resource Endowment ∩ Environmental Load Bearing	0.388	Enhancement
**I-period**	Resource Endowment ∩ Environmental Load Bearing	0.528	Enhancement
**II-period**	Resource Endowment ∩ Environmental Load Bearing	0.575	Enhancement
**III-period**	Resource Endowment ∩ Environmental Load Bearing	0.410	Enhancement

#### Driving factors of the spatial divergence across regions

Tables 9 and 10 exhibit the results of the factor detector and interaction detector for input-output on the spatial divergence across regions. The factor detector indicates that net output and non-point source pollutants were the primary driving factors for the spatial divergence of GPE in the key development region, with a q-statistic of 0.244 and 0.249, respectively. In the moderate development region, piglet input and net output had the greatest impact on spatial divergence, with q-statistics of 0.319 and 0.301, respectively. Meanwhile, piglet input had the most significant influence on the potential growth region, followed by water inputs, with q-statistics of 0.394 and 0.252, respectively. Carbon emissions and non-point source pollutants significantly affected the spatial divergence in the constrained development region, with q-values of 0.165 and 0.229, respectively.

**Table 9:** Effect of input-output on spatial divergence of GPE across regions.

Regions	Labor input	Piglet input	Feed input	Water input	Energy input	Net Output	Carbon Emission	Pollutant emission
**Key Development Region**	0.221	0.102	0.074	0.041	0.029	0.244	0.123	0.249
**Moderate Development Region**	0.178	0.319	0.127	0.077	0.225	0.301	0.112	0.204
**Potential Growth Region**	0.248	0.394	0.101	0.252	0.159	0.205	0.070	0.111
**Constrained Development Region**	0.098	0.124	0.045	0.148	0.060	0.133	0.165	0.229

**Table 10:** Interactive effect of input-output on spatial divergence of GPE across regions.

Regions	Leading interaction factor	q-statistic	Interaction
**Key Development Region**	Net Output ∩ Pollutant emission	0.495	Enhancement
	Water input ∩ Pollutant emission	0.494	Enhancement
**Moderate Development Region**	Labor input ∩ Net Output	0.727	Enhancement
	Labor input ∩ Energy input	0.711	Enhancement
**Potential Growth Region**	Piglet input ∩ Net Output	0.694	Enhancement
	Piglet input ∩ Feed input	0.687	Enhancement
**Constrained Development Region**	Energy input ∩ Pollutant emission	0.496	Enhancement
	Feed input ∩ Water input	0.485	Enhancement

The interaction detector suggests that the interaction of each factor played a major role in the spatial divergence of GPE. The interaction of net output and non-point source pollutants had the most significant effect on the spatial divergence in the key development region, with a value of 0.495. In the moderate development region, the interaction between labor input and net output was the most significant, with a value of 0.727. The largest effect of the interaction, with a q-statistic of 0.694, was between piglet input and net output in the potential growth region. Energy input and non-point source pollutants had the greatest impact on spatial divergence in the constrained development region, reaching 0.496.

Thus, it can be concluded that inputs were the primary reason for the spatial divergence of GPE in the hog breeding industry, while output status and pollution emission were the primary constraints for the green development of the hog breeding industry.

Tables 11 and 12 depict the effect of external factors on the spatial divergence of GPE in the hog breeding industry across regions. The factor detector reveals that environmental investment was the primary driver of spatial divergence in the key and moderate development regions, with a q-statistic of 0.256 and 0.145, respectively. Furthermore, resource endowment had a significant impact on the spatial divergence of the potential growth and constrained development regions, with q-statistics of 0.528 and 0.165, respectively. The interaction detector indicates that the interaction between environmental investment and environmental load bearing had the greatest impact on the spatial divergence in the key and moderate development regions, with q-statistics of 0.515 and 0.405, respectively. In the potential growth region, the interaction of resource endowment and environmental load bearing had the most significant impact on spatial divergence, at 0.714. The spatial divergence in the constrained development region was mainly influenced by the interaction between scale breeding and resource endowment, with an effective degree of 0.382. It can be observed that environmental investment and environmental load bearing were the primary drivers of spatial divergence in the key and moderate development regions, while resource endowment had the most significant impact on spatial divergence in the potential growth and constrained development regions. The interaction between these factors formed each region’s distinctive spatial divergence development pattern.

**Table 11:** Effect of external factors on the spatial divergence of GPE across regions.

Regions	Environmental Investment	Resource Endowment	Environmental Load Bearing	Scale Breeding	Production Operation
**Key Development Region**	0.256	0.094	0.138	0.150	0.106
**Moderate Development Region**	0.145	0.096	0.059	0.013	0.055
**Potential Growth Region**	0.123	0.528	0.260	0.134	0.131
**Constrained Development Region**	0.071	0.165	0.045	0.094	0.029

**Table 12:** Interaction effect of external factors on the spatial divergence of GPE across regions.

Regions	Leading interaction factor	q-statistic	Interaction
**Key Development Region**	Environmental Investment ∩ Environmental Load Bearing	0.515	Enhancement
**Moderate Development Region**	Environmental Investment ∩ Environmental Load Bearing	0.405	Enhancement
**Potential Growth Region**	Resource Endowment ∩ Environmental Load Bearing	0.714	Enhancement
**Constrained Development Region**	Resource Endowment ∩ Scale Breeding	0.382	Enhancement

## Conclusions and suggestions

The study aimed to illuminate the spatial divergence of GPE within China’s hog breeding industry and the underlying driving factors, offering valuable insights to facilitate the industry’s pursuit of high-quality development. The GPE, along with its spatial divergence, was assessed utilizing the SBM model and divergence indexes across various regions from 2006 to 2019. Subsequently, the Geodetector was employed for further analysis of the driving forces behind spatial divergence. The findings revealed a positive trend in the GPE of the hog breeding industry in China and all regions, with an average value of 0.401 during the study period. Notably, key development areas exhibited the highest average GPE, while potential growth areas registered the lowest average GPE. Previous studies, such as Wang et al. [47] employed the SFA model to demonstrate the upward trend in cost efficiency for fattening pigs, sows, and piglets, with respective values of 0.77, 0.79, and 0.53. Wu et al. [48] utilized the SBM-Malmquist-Tobit model, revealing significant inter-provincial variations in the ecological efficiency of hog production, ranging from 0.557 to 1.19 across Chinese provinces in 2018. Hence, this paper aligns closely with the findings of prior research.

The drivers of GPE within China’s hog breeding industry primarily encompass well-considered policy planning and technological advancements [46, 47]. To ensure an ample pork supply, diverse regions have devised hog development strategies based on resource allocation, industrial foundations, environmental capacities, and developmental requirements. Under the auspices of the sustainable green development paradigm, GPE has exhibited continuous advancement. However, the industry faces risks associated with epidemics and market fluctuations, which impede its stability. The promotion of large-scale breeding practices and increased investments in environmental management bolster the progression of eco-friendly breeding technologies, thereby furnishing effective safeguards against breeding risks. Moreover, these endeavors facilitate the enhancement of input-output efficiency and GPE.

Regarding temporal progression, the spatial divergence of GPE within China’s hog breeding industry undergoes a cycle of contraction followed by expansion, characterized by significant fluctuations and dispersion trends across the nation and its regions, thereby affirming the conclusions drawn in existing studies [47]. External risks, along with the industry’s limited capacity to cope with such risks, profoundly influence the evolution of spatial divergence and contribute to fluctuations in GPE [49, 50]. Unexpected occurrences like outbreaks of diseases such as PRRS and ASF, as well as social incidents like the lean meat powder scandal, trigger unforeseen fluctuations in GPE and its spatial divergence, exerting adverse effects on the stable and sustainable development of the hog breeding industry. As the backbone of China’s animal husbandry sector, the hog breeding industry’s frequent market fluctuations have garnered widespread attention. Support for the industry’s green development, encompassing capital, technology, and policy aspects, has spurred continuous growth in GPE amid these fluctuations. Furthermore, differing developmental foundations and strategic orientations across regions, as well as the formulation of distinct green development measures, inevitably contribute to the expansion of spatial divergence.

The spatial divergence of GPE witnessed expansion across all regions, excluding the key development region. The driving factors behind spatial divergence vary across different regions, predominantly influenced by resource endowment and environmental factors. While existing studies have extensively examined the influencing factors on GPE itself, exploring the causes of temporal fluctuations and the current state of GPE in various regions [46, 47, 49], limited attention has been devoted to the causes of spatial divergence, which this paper seeks to address. The findings reveal distinct driving factors contributing to GPE spatial divergence in different regions, primarily encompassing input-output factors and external influences. Among the external factors, environmental governance and environmental carrying capacity play significant roles as driving forces for GPE spatial divergence in key and moderate development areas, while resource endowment serves as the primary driving factor for spatial divergence in potential growth and restricted development areas. In terms of input-output factors, the impact of feed input and carbon emissions on spatial divergence gradually diminishes, while the influence of labor input, energy input, and net output on spatial divergence progressively increases. The disparities in various resource inputs emerge as the primary reasons for spatial divergence in GPE among regions, with output status and pollution emissions acting as crucial constraints on the green development of the hog breeding industry in each respective region.

In general, the spatial divergence of GPE in each region is primarily influenced by the regional endowment of resources and environmental factors, aligning with the conclusions drawn from previous research [12]. Hogs exhibit characteristics such as dense farming, significant emissions, and substantial governance challenges, while divergence in resources and the environment play a decisive role in determining the capacity and level of green development in hog breeding. Therefore, the future endeavor lies in addressing resource and environmental constraints, as well as bridging the gap in industrial development among regions through technological advancements and strategic planning.

To mitigate the impact of GPE’s spatial divergence on industrial development, a series of targeted measures are imperative. Firstly, the green breeding technology level needs to be enhanced, with particular focus on the research and development of labor-saving technology, material-saving technology, and emission-reduction technology, to enable the automation, mechanization, cleanliness, and efficiency of the hog breeding industry. Secondly, the key development region should combine breeding and planting, with increased investment in environmental management, optimization of livestock breed structure, and the development of hog breeds adapted to regional characteristics. Thirdly, clean and standardized hog breeding actions should be taken in the moderate development region, with acceleration in the promotion of clean technologies and equipment, and standardized breeding for reduced pollutant emissions. Fourthly, large-scale hog breeding, following the principle of "land-based breeding", should be carried out in the potential growth region, with development of large-scale, specialized, intensive breeding, and cultivation of leading enterprises. Lastly, high-end boutique actions should be carried out in the constrained development region, to extend the industrial chain, deepen product processing, and improve breeding efficiency.

However, this paper has some limitations. Firstly, it did not examine the differences in GPE among different scales of hog breeding, but instead included it as a driving factor in the analysis system to investigate the impact of scaled breeding on spatial divergence. Therefore, this paper considers the hog breeding industry as a whole and explores the spatial divergence of GPE and its driving factors. Secondly, a variety of factors at the macro, meso, and micro levels all affect the spatial divergence of GPE in the hog breeding industry. This paper integrates previous research and identifies some under-explored yet crucial industry-level factors for analysis. Future studies could select variables from multiple levels and perspectives to construct a comprehensive analysis framework of driving factors. Furthermore, due to data availability and study period, the development status of the hog industry after the COVID-19 outbreak was not fully addressed, and the epidemic’s impact on the industry requires further exploration to provide an empirical reference for the Chinese hog breeding industry to respond to external shocks.

## Supporting information

S1 Data (XLSX)

## References

[1] Wassenaar T, Castel V, Steinfeld H, de Haan C, Gerber P. Livestock’s long shadow: environmental issues and options. Renewable Resources Journal. 2006;24(4):15–7.

[2] Bai Z, Ma W, Ma L, Velthof GL, Wei Z, Havlik P, et al. China’s livestock transition: Driving forces, impacts, and consequences. Sci Adv. 2018;4(7):r8534. doi: 10.1126/sciadv.aar8534 30035221 PMC6051741

[3] Hu Y, Cheng H, Tao S. Environmental and human health challenges of industrial livestock and poultry farming in China and their mitigation. Environ Int. 2017;107:111–30. doi: 10.1016/j.envint.2017.07.003 28719840

[4] Li Y, Chen Y. Development of an SBM-ML model for the measurement of green total factor productivity: The case of pearl river delta urban agglomeration. Renewable and Sustainable Energy Reviews. 2021;145.

[5] Huang X, Feng C, Qin J, Wang X, Zhang T. Measuring China’s agricultural green total factor productivity and its drivers during 1998–2019. Sci Total Environ. 2022;829:154477. doi: 10.1016/j.scitotenv.2022.154477 35304138

[6] Yu D, Li X, Yu J, Li H. The impact of the spatial agglomeration of foreign direct investment on green total factor productivity of Chinese cities. J Environ Manage. 2021;290:112666. doi: 10.1016/j.jenvman.2021.112666 33910073

[7] Gu B, Liu J, Ji Q. The effect of social sphere digitalization on green total factor productivity in China: Evidence from a dynamic spatial Durbin model. J Environ Manage. 2022;320:115946. doi: 10.1016/j.jenvman.2022.115946 35961145

[8] Song M, Peng L, Shang Y, Zhao X. Green technology progress and total factor productivity of resource-based enterprises: A perspective of technical compensation of environmental regulation. Technological Forecasting & Social Change. 2022;174.

[9] Han Z, Han C, Yang C. Spatial econometric analysis of environmental total factor productivity of ranimal husbandry and its influencing factors in China during 2001–2017. Sci Total Environ. 2020;723:137726. doi: 10.1016/j.scitotenv.2020.137726 32213419

[10] Zhong S, Chen X, Li J, Jiang S. A perspective of scale differences for studying the green total factor productivity of Chinese laying hens. Sci Rep-Uk. 2022;12(1):6847. doi: 10.1038/s41598-022-10693-z 35478203 PMC9046397

[11] Zhong S, Li J, Qu Y. Green total factor productivity of dairy cow in China: Key facts from scale and regional sector. Technological Forecasting & Social Change. 2022;183.

[12] Zhao L, Lin J, Zhu J. Green Total Factor Productivity of Hog Breeding in China: Application of SE-SBM Model and Grey Relation Matrix. Pol J Environ Stud. 2015;24(1):403–12.

[13] Zhong S, Li J, Zhang D. Measurement of green total factor productivity on Chinese pig breeding: from the perspective of regional differences. Environ Sci Pollut Res Int. 2022;29(18):27479–95. doi: 10.1007/s11356-021-17908-2 34982382

[14] Zhou T, Jiang G, Li G, Zhou D, Qu Y. Neglected idle rural residential land (IRRL) in metropolitan suburbs: Spatial differentiation and influencing factors. J Rural Stud. 2020;78.

[15] Hongman L, Shibin W, Zhuang W. Agricultural production agglomeration and total factor carbon productivity: based on NDDF–MML index analysis. China Agr Econ Rev. 2022;14(4).

[16] Liu D, Zhu X, Wang Y. China’s agricultural green total factor productivity based on carbon emission: An analysis of evolution trend and influencing factors. J Clean Prod. 2021;278. 10.1016/j.jclepro.2020.123692

[17] Baldoni E, Esposti R. Agricultural Productivity in Space: an Econometric Assessment Based on Farm‐Level Data. Am J Agr Econ. 2020;103(4).

[18] Thomas S, Martin B, Yuanjia H. Effects of knowledge capital on total factor productivity in China: A spatial econometric perspective. China Econ Rev. 2014;29.

[19] Chapagain AK, Hoekstra AY, Savenije HHG. Water saving through international trade of agricultural products. Hydrol Earth Syst Sc. 2006;10(38).

[20] Zhaoliang L, Minghao J, Jianwei C. Economic growth of green agriculture and its influencing factors in china: Based on emergy theory and spatial econometric model. Environment, Development and Sustainability. 2021(prepublish).

[21] He W, Li E, Cui Z. Evaluation and Influence Factor of Green Efficiency of China’s Agricultural Innovation from the Perspective of Technical Transformation. Chinese Geogr Sci. 2021;31(2):313–28. 10.1007/s11769-021-1192-x

[22] He Q, Deng X, Li C, Yan Z, Kong F, Qi Y. The green paradox puzzle: fiscal decentralisation, environmental regulation, and agricultural carbon intensity in China. Environ Sci Pollut R. 2022;29(51):78009–28. doi: 10.1007/s11356-022-21149-2 35689770

[23] Li C, Shi Y, Khan SU, Zhao M. Research on the impact of agricultural green production on farmers’ technical efficiency: evidence from China. Environ Sci Pollut R. 2021;28(29):38535–51. doi: 10.1007/s11356-021-13417-4 33735411

[24] Zhu X, Chen Y, Feng C. Green total factor productivity of China’s mining and quarrying industry: A global data envelopment analysis. Resour Policy. 2018;57:1–9. 10.1016/j.resourpol.2017.12.009

[25] Zheng Z. Energy efficiency evaluation model based on DEA-SBM-Malmquist index. Energy reports. 2021;7:397–409. 10.1016/j.egyr.2021.10.020

[26] Oliver TC, Bradford LB, Graham KM, Navin R, Jean-Paul C. Leveraging total factor productivity growth for sustainable and resilient farming. Nature Sustainability. 2019;2(1).

[27] Shi R, Irfan M, Liu G, Yang X, Su X. Analysis of the Impact of Livestock Structure on Carbon Emissions of Animal Husbandry: A Sustainable Way to Improving Public Health and Green Environment. Front Public Health. 2022;10:835210. doi: 10.3389/fpubh.2022.835210 35223746 PMC8873578

[28] Yongjun L, Wenhui H, Weiwei Z, Feng L, Liang L. Provincial carbon emission performance analysis in China based on a Malmquist data envelopment analysis approach with fixed-sum undesirable outputs. Ann Oper Res. 2021(prepublish).

[29] Huang Y, Huang X, Xie M, Cheng W, Shu Q. A study on the effects of regional differences on agricultural water resource utilization efficiency using super-efficiency SBM model. Sci Rep-Uk. 2021;11(1). 10.1038/s41598-021-89293-2 PMC8113275 33976285

[30] Zhou P, Zhou X, Fan LW. On estimating shadow prices of undesirable outputs with efficiency models: A literature review. Appl Energ. 2014;130.

[31] Streimikis J, Saraji MK. Green productivity and undesirable outputs in agriculture: a systematic review of DEA approach and policy recommendations. Economic Research-Ekonomska Istraživanja. 2022;35(1).

[32] Zhang TL, Yan, Wei DM. Characteristic distribution of livestock manure and warning analysis of environmental carrying capacity based on the consumption of cultivated land in China. Chinese Journal of Eco-Agriculture. 2020;28(05):745–55.

[33] Yao CS, Qian SS, Li ZT, Liang LW. Provincial animal husbandry carbon emissions in China and temporal-spatial evolution mechanism. Resources Science, 2017;39(4):698–712.

[34] Yi Q, Kaihui S, Tao H, Tianyu Y. Environmental status of livestock and poultry sectors in China under current transformation stage. Sci Total Environ. 2018;622–623. 10.1016/j.scitotenv.2017.12.045 29223896

[35] Hu D, Jiao J, Tang Y, Han X, Sun H. The effect of global value chain position on green technology innovation efficiency: From the perspective of environmental regulation. Ecol Indic. 2021;121.

[36] Ma H, Rae AN, Huang J, Rozelle S. This document is discoverable and free to researchers across the globe due to the work of AgEcon Search. Am J Agr Econ. 2006;88(3):680–95.

[37] Liu H, Ling D. Value chain reconstruction and sustainable development of green manufacturing industry. Sustainable Computing: Informatics and Systems. 2020;28(prepublish).

[38] Yu S, Zheng Y, Hu X, Shu K. Spatial impacts of biomass resource endowment on provincial green development efficiency. Renew Energ. 2022;189.

[39] Kolleen JR, Norman R. Economic development and food production–consumption balance: A growing global challenge. Food Policy. 2010;36(2).

[40] Michelle R, Michel M, Charles HC. Is Environmental Governance Substantive or Symbolic? An Empirical Investigation. J Bus Ethics. 2013;114(1).

[41] Tone K. A Slacks-Based Measure of Efficiency. Eur J Oper Res. 2001;130(3):498–509.

[42] Haidich AB, Ioannidis J. The Gini coefficient as a measure for understanding accrual inequalities in multicenter clinical studies. J Clin Epidemiol. 2004;57(4):341–8. doi: 10.1016/j.jclinepi.2003.09.011 15135834

[43] Feng T, Zhang JY. Multicriteria evaluation on accessibility-based transportation equity in road network design problem. J Adv Transport. 2014;48(6):526–41. 10.1002/atr.1202

[44] Mookherjee D, Shorrocks A. A Decomposition Analysis of the Trend in UK Income Inequality. The Economic journal (London). 1982;92(368):886–902. 10.2307/2232673

[45] Rui Z, Liping Z, Mingxing Y, Linchuan Y. A geographically weighted regression model augmented by Geodetector analysis and principal component analysis for the spatial distribution of PM 2.5. Sustain Cities Soc. 2020;56(C).

[46] Zhu L, Meng J, Zhu L. Applying Geodetector to disentangle the contributions of natural and anthropogenic factors to NDVI variations in the middle reaches of the Heihe River Basin. Ecol Indic. 2020;117:106545. 10.1016/j.ecolind.2020.106545

[47] Wang G, Zhao C, Shen Y, Yin N. Estimation of cost efficiency of fattening pigs, sows, and piglets using SFA approach analysis: Evidence from China. Plos One. 2021;16(12):e261240. doi: 10.1371/journal.pone.0261240 34898638 PMC8668113

[48] Wu Q, Xu L, Geng X. Ecological efficiency of hog scale production under environmental regulation in China: based on an optimal super efficiency SBM-Malmquist-Tobit model. Environmental science and pollution research international. 2022;29(35). 10.1007/s11356-021-16712-2 35279751

[49] Xu G, Sarkar A, Qian L, Shuxia Z, Rahman MA, Yongfeng T. The impact of the epidemic experience on the recovery of production of pig farmers after the outbreak-Evidence from the impact of African swine fever (ASF) in Chinese pig farming. Prev Vet Med. 2022;199. doi: 10.1016/j.prevetmed.2022.105568 35008013

[50] Zhong S, Li J, Chen X, Wen H. Amulti-hierarchymeta-frontierapproachformeasuringgreentotalfactorproductivity: AnapplicationofpigbreedinginChina. Socio-Econ Plan Sci. 2022;81:17. 10.1016/j.seps.2021.101152

