# Supplementary material for: Green production efficiency of China’s hog breeding industry: Spatial divergence and its driving factors
Source: PLoS One. 2023 Nov 3;18(11):e0288176. doi: 10.1371/journal.pone.0288176 (PMC10624285; doi:10.1371/journal.pone.0288176)
Supplement: Attachment — Submitted filename: Response to Reviewers.docx [file pone.0288176.s003.docx]

**Response to Reviewers**

Dear editors and reviewers:

Thank you very much for your letter and the reviewers' comments on our paper “Green production efficiency of China’s hog breeding industry: spatial divergence and its driving factors” (Manuscript ID: PONE-D-23-04011).

We have learned much from the reviewers’ comments, which are fair, encouraging, and constructive. After carefully studying the comments and your advice, we have made a corresponding revision, and the response to the comments is enclosed at the end of this letter, with the revision contents in this manuscript marked in red. If you have any questions about this paper, please don’t hesitate to contact us.

**Reviewer 1:**

The author has indeed made significant improvements, but there are still some minor issues that need to be addressed. Here are the suggested modifications:

[Comment 1] Please verify if the data for Labor input, Piglet input, and Feed input in Table 2 and Table 3 are consistent.

**[Response]** We have conducted a thorough examination of Tables 2 and 3 to ensure the consistency of data concerning Labor input, Piglet input, and Feed input. (On Page 13 and 19)

[Comment 2] It is recommended to use the unit of US dollars for variables like Energy input and Water input throughout the entire text. Please make the necessary modifications.

**[Response]** We have applied the current year's exchange rate to convert all amounts from RMB to USD across the entirety of the text. (On Page 11, 13 and 19, in Tables 1, 2, and 3)

[Comment 3] It is advised to revise the format of Table 4. The Table currently appears unappealing and is somewhat difficult to comprehend. Please make it more visually appealing and easier to understand.

**[Response]** To enhance clarity and visual appeal, we have replaced Table 4 with Fig 1, presenting the results in a more comprehensible and captivating manner. (On Page 20)

**Reviewer 2:**

Although the two aspects of literature and writing standards still need to be strengthened, but the defects still cannot obscure the manuscript. It is therefore recommended that it be published with appropriate revisions.

The contents that still need to be modified are as follows:

[Comment 1] It is best to avoid the phenomenon of title to title. After each title, there should be a general paragraph to summarize the main content and ideas of this chapter, and explain the relationship with the previous paragraph, so as to play the role of connecting the preceding and the following.

**[Response]** First, we have scrutinized the entire text, rectifying instances where titles were used instead of headings. Second, we have appended a summary paragraph beneath each heading, succinctly capturing the main content and ideas of the chapter, while establishing connections with the preceding chapter's material.

“This chapter presents a comprehensive account of the methods and data sources employed in the research process of this paper. It elucidates the procedure for choosing indicators to measure GPE in the hog breeding industry, along with the underlying rationale for selecting the drivers. This chapter furnishes methods and material backing for calculating and analyzing the research findings in the subsequent section.” (In Lines 124~128)

“According to the arrangement of the study, this section selects the input-output variables required to measure GPE and the driving factors variables that may affect the spatial divergence of GPE to provide material support for the study.” (In Lines 150~152)

“This section provides a detailed description of the methods to be applied in this study, including the SBM model, the divergence index, and the Geodetector, which helps to improve the applicability of the study.” (In Lines 237~239)

“This chapter employs the aforementioned materials and methods to gauge the GPE of the hog breeding industry and conducts an analysis of the outcomes. It expounds upon the development characteristics and evolutionary trends of GPE in diverse regions and provinces across various time periods, examining both spatial and temporal dimensions, while offering profound interpretations for the underlying reasons. The findings of this chapter serve as the foundation for scrutinizing the spatial divergence of GPE and its driving factors.” (In Lines 310~315)

“Building upon the aforementioned measurement results, this section proceeds to delve into an in-depth analysis of the extent of divergence in GPE within various regions and provinces throughout the sample period. Expanding on this foundation, the dispersion trend of GPE divergence among different regions is explored through the implementation of an α convergence test, while elucidating the underlying reasons behind this evolving trend. Moreover, this chapter examines the distinctive features of spatial divergence in GPE within the hog breeding sector across different periods and regions, affirming the driving factors through the scrutiny of input-output factors and external factors.” (In Lines 384~391)

[Comment 2] The discussion section at the end of the article is placed in the front as the background, which is not suitable for the end.

**[Response]** We have integrated the background information discussed in the Discussion section into the Introduction, eliminating redundant content. “It not only evaluates the efficiency of resource inputs, such as land, labor, and capital but also takes environmental factors into account to comprehensively measure the overall efficiency of industries under the constraints of resource and environmental factors” (In Lines 67~70)

“This spatial divergence not only leads to an unbalanced allocation of resources and technologies and a loss of coordination in industrial development, but also affects the effectiveness of emission reduction and environmental management, resulting in social green inequity and hindering high-quality development in the industry” (In Lines 91~94)

[Comment 3] Conclusions and suggestions. Empirical results need to be further analyzed in combination with existing studies. This paper supplements the views of existing scholars in those aspects. Have any studies come to similar conclusions? If so, what does the paper add to the existing conclusions? It is suggested to combine the existing literature for in-depth analysis and discussion, not only stay in the experimental results, but also further practicallars.

**[Response]** First, we have amalgamated the "Discussion" and "Conclusion" sections into the comprehensive "Conclusions and suggestions" section.

Second, we have meticulously restructured this section, delving deeply into the empirical findings and providing thorough explanations for the observed outcomes. (In Lines 543~605)

Third, we have conducted a meticulous analysis and discussion of the results within the framework of existing literature, amplifying the credibility of this paper's findings. (In Lines 543~605)

Fourth, we have outlined the novel additions and contributions of this study to existing research, thereby enhancing the practical significance of the obtained results. “While existing studies have extensively examined the influencing factors on GPE itself, exploring the causes of temporal fluctuations and the current state of GPE in various regions [45, 46, 48], limited attention has been devoted to the causes of spatial divergence, which this paper seeks to address.” (In Lines 583~586)

**Reviewer 3:**

(No Response)

**Reference revision**

We removed the original [45]-[51] from the list of references and added the existing [45]-[48].
